# *“If You Tell People That You Had Sex with a Fellow Man*, *It Is Hard to Be Helped and Treated”*: Barriers and Opportunities for Increasing Access to HIV Services among Men Who Have Sex with Men in Uganda

**DOI:** 10.1371/journal.pone.0147714

**Published:** 2016-01-25

**Authors:** Rhoda K. Wanyenze, Geofrey Musinguzi, Joseph K. B. Matovu, Juliet Kiguli, Fred Nuwaha, Geoffrey Mujisha, Joshua Musinguzi, Jim Arinaitwe, Glenn J. Wagner

**Affiliations:** 1 Department of Disease Control and Environmental Health, Makerere University School of Public Health, Kampala, Uganda; 2 Department of Community Health, Makerere University School of Public Health, Kampala, Uganda; 3 MARPS Network, Kampala, Uganda; 4 Ministry of Health, Kampala, Uganda; 5 RAND Corporation, Santa Monica, CA, United States of America; University of Missouri-Kansas City, UNITED STATES

## Abstract

**Background:**

Despite the high HIV prevalence among men who have sex with men (MSM) in sub-Saharan Africa, little is known about their access to HIV services. This study assessed barriers and opportunities for expanding access to HIV services among MSM in Uganda.

**Methods:**

In October-December 2013, a cross-sectional qualitative study was conducted in 12 districts of Uganda. Semi-structured in-depth interviews were conducted with 85 self-identified MSM by snowball sampling and 61 key informants including HIV service providers and policy makers. Data were analysed using manifest content analysis and Atlas.ti software.

**Results:**

Three quarters of the MSM (n = 62, 72.9%) were not comfortable disclosing their sexual orientation to providers and 69 (81.1%) felt providers did not respect MSM. Half (n = 44, 51.8%) experienced difficulties in accessing health services. Nine major barriers to access were identified, including: (i) unwelcoming provider behaviours; (ii) limited provider skills and knowledge; (iii) negative community perceptions towards MSM; (iv) fear of being exposed as MSM; (v) limited access to MSM-specific services; (vi) high mobility of MSM, (vii) lack of guidelines on MSM health services; viii) a harsh legal environment; and ix) HIV related stigma. Two-thirds (n = 56, 66%) participated in MSM social networks and 86% of these (48) received support from the networks to overcome barriers to accessing services.

**Conclusions:**

Negative perceptions among providers and the community present barriers to service access among MSM. Guidelines, provider skills building and use of social networks for mobilization and service delivery could expand access to HIV services among MSM in Uganda.

## Background

In 2014, UNAIDS set ambitious targets to diagnose 90% of all people living with HIV (PLHIV), extend antiretroviral therapy (ART) to 90% of diagnosed PLHIV, and achieve viral suppression for 90% of PLHIV on ART, by 2020 [1]. Many countries have made tremendous progress towards the 90-90-90 targets. According to the 2014 UNAIDS factsheet, 10.7 million people in sub-Saharan Africa were accessing antiretroviral treatment, 41% of all PLHIV in the region, up from fewer than 100,000 people in 2002 [2]. In 2013 Uganda adopted the WHO 2013 treatment guidelines and has significantly increased enrolment of people on ART from 570,373 in 2013 to 750,896 in 2014 [3]. However, major inequities persist in access to HIV prevention, care, and treatment services [4]. Achieving the UNAIDS and national targets and moving towards elimination of HIV requires that all people at risk of HIV infection are reached equitably with quality services. In many high burden countries in sub-Saharan Africa, access to HIV services is especially limited among key populations including men who have sex with men (MSM) [4–7], despite MSM being at very high risk of HIV infection and transmission [8–10].

In Uganda, some populations have a significantly higher risk of HIV infection than others, commonly referred to as most-at-risk populations (MARPs). It is estimated that 13.7% of MSM in Uganda are HIV infected compared to the general population prevalence of 7.4% [8, 11]. HIV prevalence is even higher among older MSM (≥ 25 years) at 22.4% [8]. Despite this high burden of HIV infection and other sexually transmitted infections, access to services among MSM is low [8]. Additionally, HIV-related stigma, discrimination and the restrictive legal environment increase vulnerability and further limit their access to services [8, 12]. In 2013, the anti-homosexuality Bill was presented in Parliament of Uganda, debated and passed but was eventually repealed [3]. Access to services among MSM has not been adequately targeted and the interventions are not up to the required scale, intensity, and quality [13].

There is renewed focus towards expanding HIV services among all MARPs in Uganda, including MSM, with a growing number of service providers [13]. However, there are several knowledge gaps in terms of understanding the reach of interventions and barriers to access. The aim of this study was to explore the barriers and opportunities for increasing access to HIV services among MSM in Uganda, in order to inform HIV service programming for this population. The study specifically examines the extent to which MSM access HIV services, their experiences with accessing HIV-related prevention and treatment services, and their participation in social networks that could be used to reach them.

## Materials and Methods

### Ethics Statement

The Makerere University School of Public Health Higher Degrees Research and Ethics Committee and Uganda National Council for Science and Technology approved the study. Permission to conduct the study was also sought from the local authorities in the selected districts. For maximum confidentiality, written informed consent was done using initials of participant pseudo names, rather than signatures or thumbprints. Voluntary participation was emphasized and confidentiality maintained during interviews and throughout data handling. Soft data were transferred from recorders and stored on computers and backup files that were password protected and only the investigators had access to the passwords. Interviewer training emphasized confidentiality and respect for study participants.

### Study population and setting

**Fig 1:**
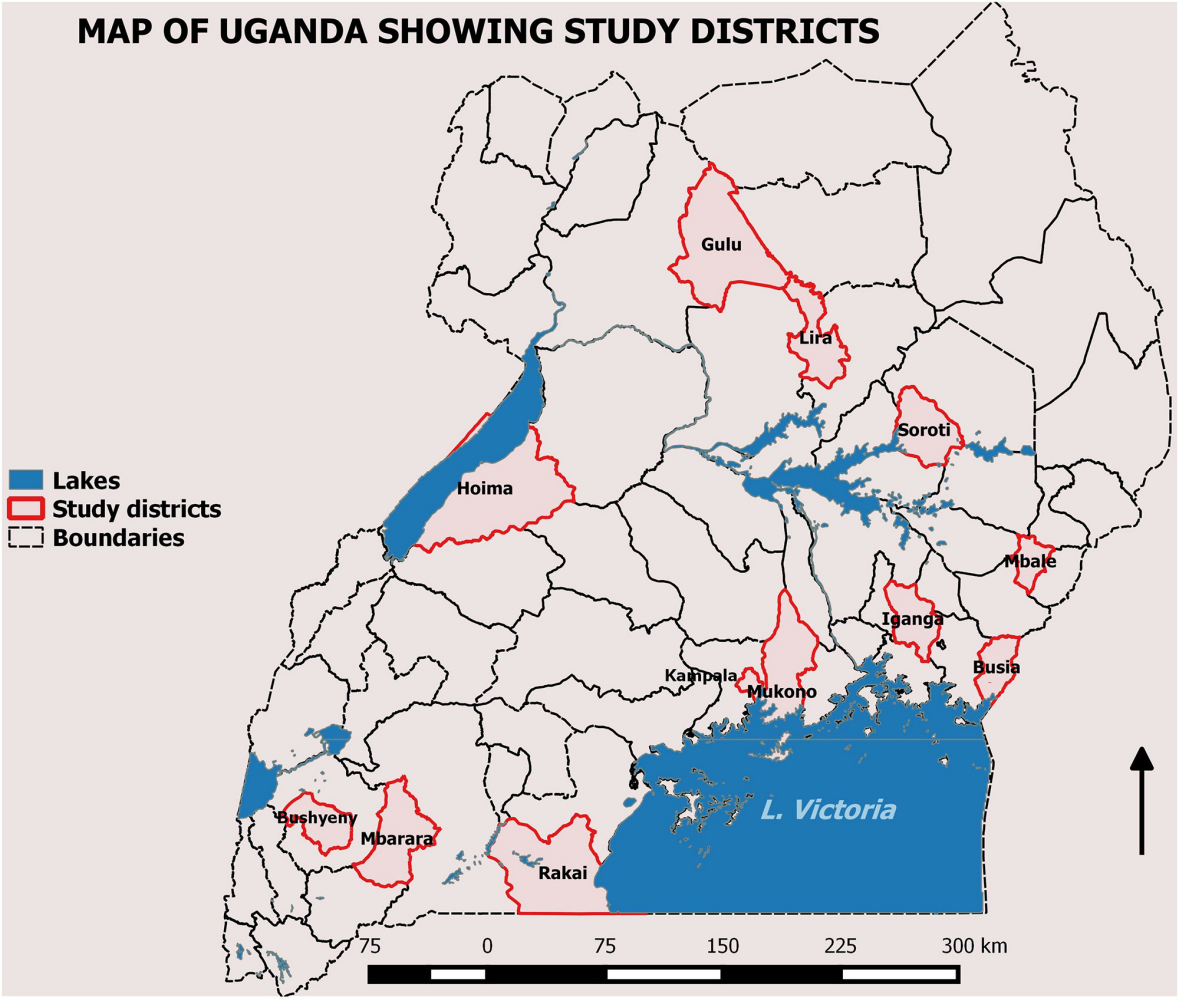
Map of Uganda Showing the 12 Study Districts. The study districts are highlighted with red boundaries.

The study was conducted in 12 districts of Uganda including Kampala, Mukono, Rakai, Busia, Iganga, Mbale, Soroti, Lira, Gulu, Mbarara, Hoima and Bushenyi (Fig 1). Selection of the districts was based on geographical representation, HIV prevalence, and known hot spots for MARPs. Most of these districts lie along the transport corridors known for high concentration of mobile and high risk populations [11].

### Participant selection and data collection

Key informants were purposively selected while the MSM were recruited through snowball sampling. The key informants were knowledgeable individuals working with MARPs, including MSM, and/or providing HIV services (Civil Society Organisations, government ministries and agencies such as Ministry of Health and Uganda AIDS Commission, and selected representatives of MSM groups). MSM were identified through social networks, civil society organizations (CSOs), and study groups that provided support to these populations. Using three national level seeds, at least one seed was identified in each district, as an entry point, and participants were recruited by the snowballing technique. The secondary seeds within each district identified respondents within the districts. Face-to-face interviews were conducted with all respondents using semi-structured interview guides and qualitative data were audio recorded.

The interview guides were pretested and rigorous one-week training conducted to standardise procedures. The training entailed a review of the study objectives, interviewing techniques with emphasis on special issues among key populations, and detailed instruction on administering the interview guides. The training also addressed the approach to the communities and selected community members participated in the training to enhance understanding of the target community. Some of these community members were recruited as seeds and thoroughly briefed about the objectives of the study and the importance of selecting appropriate participants. All self-identified MSM were eligible for recruitment and there were no additional qualifying criteria. The investigators closely supervised data collection and conducted some interviews.

### Measures

The semi-structured in-depth interviews addressed: 1) socio-demographic variables such as age, education, work, religion, and relationship status; 2) participation in social networks and sexual behaviors such as condom use, and type of partners; 3) access to services such as HIV testing, access to and utilization of condoms and lubricants; 4) barriers to access of HIV and other health services; and 5) opportunities and recommendations for improving access to HIV services among MSM (e.g. structures and/or social networks for MSM, preferred models of service delivery). Open-ended questions were used to explore new and unexpected leads, and generate rich personal experiences and narratives. Answers to open-ended questions were followed by standard probes and closed-ended questions. Data related to use of condoms and lubricants is reported elsewhere and is thus excluded from this paper [14].

Key informant interview guides addressed several themes including: 1) Access to HIV services; 2) successes and challenges in providing HIV services for MSM; and 3) how they felt about providing services to MSM, 4) barriers and opportunities for improving access to services (e.g. existing policies and protocols that may hinder or enhance access to services by these groups). All respondents (key informants and in-depth interviews) were asked about the potential impact of the legal environment on access to services by MSM. The selection of themes for the interviews was guided by previous literature on barriers to HIV service access among MSM in Uganda and other contexts.

### Data management and analysis

Data were transcribed verbatim and concurrently translated from the local languages into English. Each transcript was reviewed by at least two individuals from the research team who were fluent in both English and the local language. Data were organized with the help of Atlas.ti version 7. Structured questions were managed and analyzed using SPSS version 17 software.

Qualitative data were analysed using manifest content analysis techniques [15–17]. Description of the visible, obvious components of text and what the text says in the transcripts was taken into account in the coding process. The text was divided into meaning units and then reduced into condensed meaning units. Next, codes were extracted from each condensed meaning unit and grouped into sub-themes and themes. Data was pile sorted to identify key emerging themes. The investigators generated the themes by examining similarities and differences between codes [17]. Rich textual quotes that reflect the meaning of selected themes were identified and integrated into the results.

## Results

Overall, 61 key informants from 12 districts and 85 MSM from 11 districts were interviewed. As shown in 1, the majority of the MSM (n = 58, 69.4%) were below the age of 25 years (mean = 24.2 and standard deviation 4.2), had primary or secondary level education (n = 56, 65.9%), and belonged to the Catholic (n = 34, 40%) or Muslim (n = 24, 28.2%) religious affiliations. Nearly two-thirds (n = 55, 64.7%) were employed; three-quarters (n = 65, 76.5%) were in sexual relationships with men while 14 (16.5%) were in sexual relationships with women (Table 1). The 61 key informants who were interviewed included 48 from the districts. Four individuals were interviewed from each district including the district health officer or the HIV focal person, two providers from a public health facility providing HIV services (a nurse or counsellor and doctor) and one provider from an NGO facility proving HIV services. The rest of the key informants were national level policy makers, development partners, and representatives of MARPs groups including MSM networks.

**Table 1:** Characteristics of the in-depth interview respondents.

Variable	N (%)
**Age**	
18–20	16 (18.8)
21–25	43 (50.6)
>25	26 (30.6)
**Education**	
None	10 (11.8)
Primary	27 (31.8)
Secondary	29 (34.1)
Tertiary	19 (22.4)
**Religion**	
Catholic	34 (40)
Anglican	20 (23.5)
Muslim	24 (28.2)
Seventh Day Adventists	6 (7.1)
Pentecostal	1 (1.2)
**Employment status**	
Employed	55 (64.7)
Not employed	30 (35.3)
**Marital status**	
Married/cohabiting (male partner)	11 (12.9)
Married/cohabiting (female partner)*	8 (9.4)
In a sexual relationship with male partner	65 (76.5)
In sexual relationship with female partner*	14 (16.5)
Never married/never cohabited	14 (16.5)

*Note: All the 14 respondents in sexual relationships with female partners (except 2) were also in sexual relationships with male partners. Among the 8 who were married or cohabiting with female partners, 6 were also in sexual relationships with male partners.

### Access to HIV services

Overall, 76 (89.4%) of the MSM ever tested for HIV, 52 (61.2%) used a condom at last sexual intercourse and 64 (75.3%) used a lubricant at least once in the previous 12 months. Half of the respondents (44; 52%) reported difficulties in accessing health services. Respondents reported that lubricants were more difficult to access in rural than the urban districts [12]. Two-thirds of the respondents (56; 66%) participated in MSM social groups and 86% of these (56) reported receiving psychosocial and material support from the networks, to overcome barriers to accessing HIV services (Table 2).

**Table 2:** Perception scale of HIV services among MSM.

	Scale N (%)		
Item	1	2	3
I am not comfortable explaining my sexual practices and reproductive health illnesses to health workers	62 (72.9%)	3 (3.5%)	20 (23.6%)
Health workers would not pay attention to me if they knew I was having sex with another man	56 (65.9%)	12 (14.1%)	17 (20.0%)
There are no HIV services that address MSM special health issues	56 (65.9%)	10 (11.8%)	19 (22.3%)
Health workers are rude and do not respect MSM	69 (81.1%)	5 (5.9%)	11 (13.0%)
If I was seen by my relatives’ friends or neighbors seeking MSM-specific HIV services, they would consider me an outcast	73 (85.9%)	3 (3.5%)	9 (10.6%)

Perception scale: 1 = strongly agree/agree, 2 = neutral, 3 = disagree/strongly disagree

### Barriers to accessing HIV and other health services among MSM

Table 2 summarizes the perceptions of MSM towards a range of factors that affect their access to health services. Overall, 62 (72.9%) agreed or strongly agreed with the statement “*I am not comfortable explaining my sexual practices and related illnesses to health workers*”; 56 (65.9%) felt that health workers would not attend to them if they knew they were MSM; 69 (81.1%) felt that health workers were rude and did not respect MSM. Overall, 44 (51.8%) had experienced difficulties in accessing health services. The main challenges included stigma associated with being MSM (n = 15), failure to afford care (n = 15), and lack of personnel to handle MSM special care (n = 12).

Qualitative data yielded nine major barriers, including: (i) Negative attitudes and unwelcoming behaviors of health workers and the health care environment; (ii) Health care workers’ lack of sufficient skills and knowledge to manage MSM-specific health care needs; (iii) Negative community perceptions towards MSM; (iv) fear of being segregated or exposed as MSM; (v) limited access to MSM-specific services; (vi) high mobility of MSM population, (vii) lack of national-level guidelines on how to deal with MSM; viii) a harsh legal environment; and ix) general fears related to HIV-associated stigma and HIV testing. The findings from each of these sub-themes are further described below.

### Study design and sample size

This was a cross-sectional study that employed qualitative methods using semi-structured in-depth interview guides and key informant interview guides. Overall, 85 in-depth interviews were held with self-identified MSM in 11 districts and 61 key informants in 12 districts. The key informants included 48 district and 13 national HIV and other health service implementers (public and private).

### Negative attitudes, unwelcoming health workers and the healthcare environment

The behaviors and attitudes that MSM considered unwelcoming included subjecting them to many questions and “*bad*” comments. Some of the offensive questions from health care workers included: *Why are you doing this*? *Why do you have sex with other men*? *Don’t you want to have children*? *When will you stop this*? They reported that some health workers “*gossip”* about them (e.g. their dressing and hairstyles) and consider them to be abnormal while others don’t want to provide them with services.

“*Sometimes when you are conversing you hear them [health workers] saying that now look at that one the way he’s walking may be he’s gay and they go on saying bad things about gays*. *I just also contribute to the conversation so as not to feel out of place*. *You see these rural places most of the people condemn it [gay]*” (IDI Bushenyi).

Some MSM reported that when health workers learn that they are MSM, some deny them care while others delay to attend to them and instead prioritize the “*straight people*” because they are not comfortable working on MSM.

*“I had an infection underneath my private parts; I had a rotten part [wound]*. *But health workers never wanted to touch me*. *Men had raped me and I was rotting underneath; I got so scared to go to any health facility because people were telling me no one will touch you because you’re a gay*, *until I went to Mulago and I underwent surgery*. *When I reached Mulago no health worker wanted to touch me; everyone was asking what happened to this one until we forged a statement that we took alcohol and my friend raped me but I had rotted and I couldn’t walk for something like three or four months” (IDI Kampala)*.

Several respondents particularly feared to tell health workers about symptoms of STIs especially if they had anal lesions and/or discharge. It was more difficult for them when the provider was a woman.

*“Disclosing*, *telling the doctor that word is hard*. *It may be easy to you but not to me to tell it to a doctor*, *in other words that I have this anal problem…* *I may be shy*. *Many attendants in most drug shops are ladies and you may feel shy to tell her that you have [anal] gonorrhea” (IDI Hoima)*.

### Fear of being exposed as MSM: MSM and provider perspectives

This theme yielded a lot of fears among those who had not disclosed their sexual orientation to their providers while those who had done so had mixed experiences. Respondents feared to disclose because they did not trust their providers. They reported several negative expectations including: society would curse them; the providers would call police to arrest them; they would tell the whole community; among others. However, some were not sure and did not want to take any chances while others pretended to be “straight” since they could not predict the reactions of the providers if they told them they were MSM. Some of the men feared that disclosing their sexual orientation could cause a lot of problems including job loss, being considered an outcast, being attacked by the community, and loss of dignity.

*“I didn’t [disclose] because I feared to be attacked by the public and by the government and I feared losing my job and my dignity in public*, *losing my wife*, *my friends and the rest like you know really*. *I have a child who calls me father*, *now an old girl of primary seven; would she still call me father*? *I mean she would not call me father again” (IDI Bushenyi)*.

Whereas those whose providers did not know had very negative expectations, some of those whose providers knew did not experience such severe discrimination. Some said to their surprise, their providers never treated them badly.

*“He was actually accommodative; I was also surprised” (IDI Soroti)*.

*“He told me that; well*, *if I am MSM*, *I should endeavour to have one partner*. *He also told me that he has treated some other young men who were MSM” (IDI Rakai)*.

On the contrary, several HIV positive MSM said they felt degraded, embarrassed, and others felt ignored. Participants reported higher levels of stigma and discrimination in public facilities especially the lower level health facilities in the rural settings.

*“There is a friend of mine who went to the same facility … they told him to go with a partner and when he went with his male partner*, *they asked what are you guys doing*?* … so*, *they just left” (IDI*, *Kampala)*.

Health providers also noted that their patients may not freely open up to them and they did not ask about one’s sexual orientation when providing services. Moreover, service providers noted that unless there is a reason, it is not always possible or necessary to ask for one’s sexual orientation.

*“The biggest challenge is that these people don’t expose themselves unless you accept them [eat*, *drink*, *talk and sit with them]*, *it’s when they will be open to you but when you try to rank yourself in a different way they will also not be open to you” (District KI)*.

Several providers who were open to providing services to MSM noted that they needed to help because some of them were “innocent”.

*…the young boys we have encountered are really innocent*. *They went for MSM because some people are offering them fees*, *some people offered them jobs*. *Like they went there to meet ends*, *it wasn’t their calling but they found themselves into it)*. *(District KI)*.

### Lack of MSM specific services and skills among providers in some districts

There was a general perception among MSM that some health workers don’t have the skills and knowledge to handle MSM related issues and find it difficult to provide services to them. They reported that most districts lacked the specialised personnel and relevant services and they occasionally travelled to Kampala for such services.

*“…I told the doctor that I am MSM and he told me that it was his first time to hear about MSM and he had no knowledge of handling issues related to MSM*. *He told me that he did not know anything about MSM*, *he was not taught about it at medical school” (IDI Kampala)*.

Some health workers also acknowledged that they lacked the skills to provide services to MSM and several had never interacted with MSM. When asked about how comfortable they would be, to serve MSMs, some expressed a lot of discomfort.

*“…when I see my fellow man doing that kind of thing—I would feel very low indeed and I for one I wouldn’t encourage a man to do that kind of thing” (District KI)*.

Several providers felt it would be good to receive relevant training in order to provide quality services to MSM. However, others did not even want to receive the training.

*“I feel I am missing that training of handling sex workers who are many in our country*. *With homosexuals*, *I don’t think I would be willing to go for that training” (District KI)*.

### Community stigma and its impact on access to health services

According to the MSM, the community plays a significant negative role in preventing them from accessing services and disclosing their sexual orientation to health providers. They reported strong homophobia in the community and felt that it was not a good idea for them to reveal their identity. Participants also indicated that fellow patients make them feel uncomfortable to seek services because of the negative body language and talk.

*“You are seated with so many people*, *and you know the way you look*. *People are looking at you*, *people are laughing at you*, *and you face all those problems*. *Then sometimes you can even fail to get treatment” (IDI Kampala)*.

Whereas most MSM noted that providers had stigmatising attitudes towards them, most providers felt the stigma and discrimination was arising from the communities.

*“The stigma is created by the general community rather than the facility itself and clients do not clearly distinguish the general public from the facility because for them when they see health workers*, *they see people*, *they do not know whether they can trust that health worker that much” (District KI)*.

### High mobility of MSM population

Both the providers and MSM noted that MSM, especially the male sex workers, were as mobile as the female sex workers. This poses a challenge with regard to retention and adherence to services and medications, especially for those who are HIV infected. To ensure retention health workers provide them with counseling.

*“…the fact that they are mobile causes a challenge especially if they are on treatment but what I think is helping is to make them know that it’s their life and they have to manage it so if you go to an area where there’s no treatment and your three months are gone your drugs are over*, *you have to go back and refill and I think that’s the message we’re giving them” (National level KI)*.

The in-depth interview respondents cited several reasons for failure to return to their clinics for follow-up treatment including lack of money, long distance to health facilities, and failure to obtain permission from their employers.

### Inadequate policy guidance on MSM services and harsh legal environment

This theme yielded mixed views across various categories of respondents. Several central level key informants felt the legal environment was not a major hindrance and noted that they were using a “public health approach” which was in line with “universal access” provisions of the national policies while others felt the legal environment was a hindrance to service access. The providers also had equally mixed views. Some providers felt the legal environment does not necessarily prevent the MSM from accessing health services and instead limits activities related to their sexual practices. On the other hand, others said the environment was restrictive and they would be uncomfortable providing services to MSM if the Bill in Parliament was passed into Law. Several providers and policymakers also felt the guidelines on how to provide services to key populations, particularly for the MSM, within the existing legal context were inadequate and recommended revision and/or development of guidelines to support implementation. Majority of the providers and some central level respondents indicated that while there was guidance on how to deal with sex workers, there was no policy pertaining to MSM. They noted that MSM were only mentioned in the National HIV and AIDS Strategic Plan as a key population but not much guidance was provided on how they should be reached and what services should be provided to them.

The MSM expressed more discomfort with the legal environment than the providers and policymakers. When asked how the current legal framework affects their ability to seek health services, MSM reported that the legal environment makes them fear to access services or disclose their identity to providers.

*“If you tell people that you had sex with a fellow man*, *it’s hard to be helped and treated*. *Government does not permit homosexuality*, *indeed it is a serious crime*, *if found you are arrested” (IDI Eastern Uganda)*.

They also noted that the legal environment made it difficult for organizations that support them to print any MSM specific education materials and this hampers any efforts to provide health education and information to them. Although not frequently mentioned, some HIV positive MSM reported that they had been imprisoned on several occasions due to their sexual identity and missed their medications, which they had left behind yet drug refills were not possible within the prisons. The MSM indicated that the passing of the anti-homosexuality Bill into law would make health providers fear to provide services to them. Participants across several districts felt it would further affect disclosure of sexual orientation to providers.

### General health care access challenges and HIV related stigma

Beyond the MSM-specific barriers, other challenges to accessing HIV services were reported. Receiving HIV positive results was reported as shocking, and an experience that causes fear, self-devaluation, anger, blame, and shame. Consequently, some MSM feared taking an HIV test. Beyond the stigma associated with being MSM, HIV infected MSM suffer a second layer of stigma associated with HIV and some feared to access treatment at health facilities and to pick their drugs especially from locations that are known to provide HIV services. Several challenges with public health facilities were raised, including long queues; drug stock outs and some reported resorting to private facilities. However, lack of money was also raised as a barrier to accessing private health services.

### Misinformation from social network leaders and members

Despite the positive role of social networks in addressing barriers to accessing HIV services, concerns arose about the messages from some group leaders and members, which were potentially harmful in relation to HIV transmission and homosexuality. For example, one message that emerged from three districts including Iganga, Rakai, and Hoima was that being a homosexual protects one from acquiring HIV, and it was safer than having sex with a woman.

*“We used to talk about HIV even though majority of my colleagues always said one can never contract HIV after having sex with a fellow man*, *they used to say HIV is only acquired if someone has sex with a woman” (IDI Hoima)*

*"They tell us during trainings that when you have sex with a fellow man you can’t get any infections*, *those are the teachings we get from our colleagues who train us and even they tell us that you can only get infections when you have sexual intercourse with a woman*. *(IDI Iganga)*

### Opportunities for increasing access to services among MSM: MSM and provider views

This study identified a number of opportunities that could be utilized to extend services to MSM populations. The opportunities can broadly be classified into: enabling policy provisions, expanding existing service delivery models such as the Most at Risk Population Initiative (MARPI) model, leveraging the private sector and civil society, using social networks for education and mobilisation for service uptake, use of social media and telephone, and integration of MSM services in the outreach HIV programs.

### Enabling policy environment

Despite the legal restrictions and fears that were raised by respondents, some policymakers and providers felt there was an opportunity for providing services to MSM and cited relevant policy frameworks that provide for universal access to health services. They cited various efforts to increase access to HIV services among MSM populations including the MARPI clinic that provides facility and community-based services for sex workers and MSM. They noted that efforts were underway to expand the program to other hospitals in the country. There were also a number of NGO facilities providing MSM friendly services.

*“When you look at the services that we provide here under MARPI clinic*, *they are for the key populations only that the focus is on the MSM and the female sex workers and you know for these people its linked and they are the two populations that are finding it hard to access services from the general public health facilities because of the stigma and discrimination (National level KI)*.

The MSM also cited an appreciated the MARPI model as well as several NGO and private-for-profit organizations that provided services to them, in addition to support from their networks.

### Use of MSM social networks and telephone to improve access

MSM were asked about social networks and the extent to which they participated in such networks. Overall, 56 (66%) participated in MSM social networks or groups. Of these, 48 (85.7%) reported that the groups helped them to address challenges in accessing HIV prevention and treatment services. However, most MSM social networks were based in Kampala and the surrounding districts, and thinned out beyond Kampala (14 groups were cited in Kampala, four in Gulu, three in Hoima, two in Rakai, Mbale, and Iganga; one in Mbarara, Bushenyi, and Busia, and none in Soroti). In some districts, such as Busia, Iganga, Mbarara, Hoima, and Gulu, the Kampala-based networks had volunteer liaison contact persons. Some of the upcountry districts had their own informal groups including one in Gulu and another in Mbarara.

Besides the social network groups, social media such as Facebook, Twitter, and telephone applications were popular as network tools both at individual and community level. Some MSM had formed Facebook and Twitter groups. The composition of social networks was varied. Whereas some networks had only MSM, or only trans-women, others had both MSM and transgender.

*“To be a member*, *it requires that you are not going to spy on us and you have to be a Kuchu [MSM] and you know the problems Kuchus go through*. *If you are from a Kuchu organization and you are not a Kuchu*, *we also become suspicious” (IDI Kampala)*.

Although social networks varied in organization and structure, one role they all had in common was information sharing. In some places especially in the city, social networks acted as a source of security and belonging. More organized groups particularly in Kampala, conducted mobilization, regular meetings, linked members to health services, provided psychosocial counselling, and sometimes-material support. The Kampala networks were actively involved in negotiating with service providers and linking their members to health providers through dialogues, scheduling appointments as well as ensuring that services were extended nearer to their communities through outreach programs. One respondent in Kampala named four organizations they had linked up with, that can provide MSM friendly services.

*“…they [network leaders] have dialogues with medical practitioners telling them about LGBT issues and those medical practitioners have actually become very positive towards LGBTs and how to handle them in health related issues” (IDI Kampala)*.

Some networks sensitize their members to practice safer sexual behaviours. They encourage them to use condoms and lubricants and in case their members are sick, they encourage them to seek care and treatment. Social networks were also involved in distribution of condoms and lubricants in addition to community sensitization.

*“They always give us advice for instance when you are with your partner protect yourselves and inform the rest who don’t know that when you are in such an act when your partner has HIV and you haven’t tested*, *you should go and test in order to know if both of you are HIV negative or HIV positive so that you start on ARVs” (Mukono IDI)*.

MSM social networks in Kampala and to a lesser extent in rural districts were engaged in providing some services to the community. For example in Kampala, a number of them were spearheading HIV prevention campaigns among MSM and one organization had a clinic for LGBTI. Some respondents argued that it would be easier for MSM to access services from LGBTI providers. They felt a gay doctor would be more understanding and provide good care.

Social media and telephone were used in the communities for social networking and some MSM occasionally posted health-related messages on Facebook and Twitter. Some MSM suggested telephone and text messaging as viable communication channels through which members can be notified about health services. KIs also cited a facility that had integrated toll-free telephone lines for the communities. However, some participants did not like these channels, fearing that they might expose them to people who do not know that they are MSM. One of them (a bisexual respondent) said that such communications would create problems especially with his wife. He was concerned that his wife would read the message and know that he is a homosexual.

### Training and orienting healthcare workers to MSM healthcare needs

Despite the legal impediments and the strong feeling by MSM that health workers discriminate against them, some health workers were accommodative and were ready to provide services to MSM. However, several key informants noted the limited capacity and inadequate training among providers and recommended training and mentoring to address the knowledge gaps and attitudes. The MSM noted that most healthcare providers don’t understand MSM issues, arguing that MSM face difficulties addressing their health needs to health providers who don’t understand them. They reported that when they seek health care from well-trained and friendly providers, they feel comfortable explaining their issues.

*“I think doctors in each and every hospital should get conferences to educate them that amongst the people you receive*, *you have to receive an LGBTI at least one per day even if you don’t know them*. *Like; teaching them or telling them that these people or these things happen and they are there*. *So that if he is a doctor he will get to know” (IDI Kampala)*.

### Models of MSM service delivery

Some IDI respondents recommended MSM-only facilities arguing that the discrimination against them would be less. However, many MSM were sceptical about MSM-only facilities and recommended enhancing current services and facilities to provide MSM-friendly services. They were concerned that isolating MSM would create more exposure, stigmatization and discrimination. Because of that, some MSM felt MSM-only facilities should be disguised and that public facilities with modifications would be the best option. Nevertheless, they cited one MSM-only Clinic that was already operating.

*“It can work but it can never be 100% effective because there are those who fear*, *they are like how will they see me entering such a facility*? *Because everyone will know that at XX’s place it’s where homosexuals go for health services so this person will get scared of going there” (IDI Hoima)*.

*“In Uganda it will take years to start up MSM-only health facilities but integrating these services in existing health facilities I think it will be easier” (IDI Iganga)*.

### Outreach services

Respondents noted that some civil society organizations (CSOs) were providing services for MSM and other most-at-risk groups and could be supported to expand access to MSM friendly services. They also noted that some organizations were willing to extend services to MSM but had difficulties in penetrating the communities. Participants felt that some minor modifications in outreach programs at these CSOs could extend services to the MSM populations. For example, they suggested inclusion of a mobiliser (preferably one who is MSM) to mobilize communities and support outreach programs.

*“…if you know someone is a fellow Kuchu [MSM] you become confident*. *You have full trust in this person and he is also able to tell you everything (IDI Hoima)*.

#### Expanding services in public facilities

Although highly criticized for not being welcoming, some respondents (both MSM and providers) felt that the government health facilities were cushioned against legal impediments surrounding provision of services to MSM. In addition, since government facilities were spread all over the country, and they argued that these facilities needed minor modifications to cater for the MSM specific needs. Reference was made to Mulago hospital, which had a specialized facility for key populations, including MSM.

*“…it’s good that they know that we are there and we are citizens of Uganda*, *we pay taxes*, *and contribute to community and national development*. *Then they have to provide us with at least a small clinic in each and every big hospital per district*. *They should provide a small clinic for the LGBTI” (IDI Kampala)*.

### Use of private facilities

Participants noted that the private facilities provide an opportunity to quickly expand access to MSM friendly facilities. This was predominantly advanced by the MSM who tended to prefer private facilities to government facilities for quick services, having medicines readily available and having less patient load.

### Friendly health facilities

To explore more opportunities for increasing access to services, we asked MSM to mention what made facilities welcoming or friendly to MSM and they mentioned several features, most prominently “*welcoming providers who are trustworthy*”. A health facility was considered welcoming if staff were friendly, were not discriminating against clients based on their sexual orientation and offered prompt services. According to the MSM respondents, most public health facilities didn’t measure up to this standard unlike the private clinics and research facilities. Providers were also trustworthy if they could maintain confidentiality. They revealed that providers who were welcoming were also trustworthy and friendly. This provided an opportunity to build trust within the MSM community and could potentially encourage them to open up to some providers. Even where entire facilities may not have an orientation towards MSM friendly services, they noted that some individual providers were welcoming and supportive.

*“Actually they [friendly facilities] are not there in Gulu but there are professionals who work with both the private and the public facilities who now understand our [MSM] problems; the facilities themselves do not understand us but those individuals whom we have made contact with understand us and what we go through” (IDI Gulu)*.

### Factors influencing choice of facility by MSM

When MSM were asked what they considered important when choosing a healthcare facility for HIV services, a number of factors emerged. These included the location of the facility and the nature of sickness, facility factors, attitude of the providers, approval by peers, and existence of service providers who understand the health care needs of MSM. Participants preferred far-off facilities to reduce chances of meeting people who are known to them, minimise the likelihood of exposure of their sexual identity, and the resultant stigma. In addition, the nature of sickness contributed to care seeking decision. For minor sickness, “*usual*” nearby places would be used but if the illness required more privacy (e.g. sexually transmitted infection), then friendly providers and far off facilities were preferable.

*“I go to him every time I and my wife get fever whether during pregnancy or anything*. *So this is our family doctor*. *He is an old man who even used to treat us when we were still young and he was paid by our father*. *Therefore am free with him*. *However if it is a private sickness and I do not want him to know*, *I go to another friend of mine I told you about at KIU hospital in Ishaka” (IDI Bushenyi)*.

Some MSM indicated that they prefer to seek healthcare from facilities with providers who understand MSM health care needs. This increases their level of comfort to disclose their illnesses and also helps to reduce stigma.

*“In these facilities [with providers who understand MSM health care needs] when you go there you explain everything you feel and any illness you have the way it is; you say everything openly and you put all your issues on the table” (IDI Kampala)*.

MSM reported that they preferred private to government facilities because private facilities have fewer patients and provide confidentiality, and tend to be caring because of the financial incentive. Moreover, private facilities are better equipped with the necessary medicines.

*“Because private facilities don’t have as many people as government health facilities so this will help me keep my confidentiality as chances of meeting people I know of will be low” (IDI Rakai)*.

Because of the stigma, many MSM first seek approval from their peers, member organizations, and networks before going to a health facility. Many seek care from certain facilities that they regard as friendly. Such facilities are recommended to those seeking care by the networks.

*“There is a time I had an STI and I could not go to these public health facilities*, *so there is some friend of mine who is in the Netherlands whom I talked to and he referred me to that lady*. *The lady has been so good to me she has treated me and she has even been supplying me with lubricants and condoms” (IDI Kampala)*.

## Discussion

This study assessed barriers and opportunities for expanding access to HIV and other services among MSM and reveals many challenges in accessing services, including; negative provider attitudes and behaviours towards MSM, limited skills among providers to address MSM health needs, limited community engagement and miss-information within MSM networks, and fears related to the restrictive legal environment and community stigma. Opportunities for increasing access to HIV services among MSM include existing social networks for demand creation and delivery of health services, existence of “friendly” providers and programs, use of innovative approaches such as telephone and social media for information dissemination, and models of service delivery that can be replicated.

Several of these barriers have been reported by other studies among MSM in sub-Saharan Africa [18–21]. However, our study goes a step further to identify opportunities to deliver services to the MSM despite the prevailing environment. If not addressed, the challenges identified in this study could limit access to quality health services, compromise client-provider relationships [19] and may lead to delay or avoidance of HIV services by MSM [20]. MSM may also be less inclined to disclose their sexual orientation and other health-related behaviors to inform subsequent clinical decisions [12]. In our study, MSM experienced stigma within the communities and facilities (ranging from the negative body language to intrusive questions and rude remarks), were living in fear of the reactions from the communities, and many feared to disclose their sexual orientation to their providers.

Both MSM and providers highlighted skills gaps among providers [22, 23]. The provider attitudes including respect, non-discrimination, and politeness as well as confidentiality featured prominently in the description of “friendly” services by the MSM. Unfortunately some providers were not open to receiving training in MSM related care. Yet, the clinical curricula in low- and middle-income countries do not address these knowledge gaps. Provider skills gaps have been highlighted in several studies and innovative online training approaches have been tested in some countries, including Kenya [23, 24]. In the long-term emphasizing medical ethics, including providing services to all without discrimination through in-service and pre-service training will be important. Our findings, however, also identified gaps in the processes including policy guidance and procedures as well as standards and definition of packages for services among MSM. Several international guidelines exist, that can be useful [10, 25] but local context specific adaptation is necessary to drive implementation.

Homosexuality is a taboo in Uganda and amongst many African countries [20] and stigmatization and discrimination are prominent [8]. Thus, HIV positive MSM experience double layers of stigma (MSM related and HIV related) [26, 27]. This is not surprising since HIV related stigma still prevails in sub-Saharan Africa [27]. There were a lot of underlying fears among MSM, although some of those who had disclosed to their providers did not have negative experiences. To overcome these fears, it will be necessary to integrate counseling and confidence building interventions for the communities in tandem with provider training. Use of patient navigators as suggested by Ross and others could help to not only navigate such challenges in accessing care but could also go a step further in mobilizing communities to demand and access services [28]. Our findings indicate that involvement of the communities to identify such “Navigators”, preferably community members, might be more acceptable to the communities.

Despite the challenges and gaps in services for MSM, this study reveals a number of opportunities for expanding services to MSM, highlighting both demand and supply issues and opportunities. Our findings reveal various opportunities, including existing public and especially private facilities and MSM social networks that were already providing such services. These provide a good starting point to pilot and scale up innovative interventions for MSM. MSM networks provide an opportunity for demand creation and service delivery [7]. However, the networks were not well developed outside Kampala and some perpetuated messages that were counterproductive to HIV prevention. Identifying these groups and enhancing their capacity to deliver correct information may increase opportunities for HIV-related messaging and distribution of prevention commodities such as condoms and lubricants. More effort will be required to extend the reach of current services to the rural underserved areas and a few friendly providers. The network of partners providing services for sex workers could integrate MSM services.

Study Limitations: The findings of this study may not be generalizable to all MSM in Uganda because the sample selection through known MSM networks could have biased the study towards MSM who were participating in networks with better access to services. However, an attempt was made to encourage the participants to identify some MSM who were known to them and did not participate in social networks (34% of the respondents in this study did not participate in social networks). Also, we did not document the response rate (how many potential respondents the seeds approached and how many of these may have declined to participate in the study). Despite these limitations, the study highlights key barriers and opportunities that could be utilized to scale up access to HIV services among MSM in Uganda, and other settings with high levels of community stigma and discrimination against MSM.

In conclusion, this study highlights several gaps and challenges in the implementation of HIV services for MSM including demand (appropriate messaging and community mobilization), gaps between policy and implementation especially the lack of guidance and procedures (processes), quality concerns in relation to client-provider relationships due to negative provider attitudes, confidentiality concerns and failure to disclose sexual orientation as well as risky sexual practices. Comprehensive interventions that address the demand and supply of services for MSM are required; demand creation through the community networks, stigma reduction and concurrently ensuring availability of quality friendly services would bridge the current service gaps in Uganda.

## References

[1] Joint United Nations Programme on HIV/AIDS (UNAIDS). October 2014. 90-90-90 An ambitious treatment target to help end the AIDS epidemic. www.unaids.org/sites/default/files/media_asset/90-90-90_en_0.pdf. Accessed 5th June, 2015.

[2] Joint United Nations Programme on HIV/AIDS (UNAIDS). 2014 Global Statistics. http://www.unaids.org/sites/default/files/en/media/unaids/contentassets/documents/factsheet/2014/20140716_FactSheet_en.pdf. Accessed 14th December, 2015.

[3] Uganda AIDS Commission. The Uganda HIV and AIDS Country Status Report for 2014 http://www.unaids.org/sites/default/files/country/documents/UGA_narrative_report_2015.pdf. Accessed 14th December, 2015.

[4] WHO, Prevention and treatment of HIV and other sexually transmitted infections among men who have sex with men and transgender people Recommendations for a public health approach 2011. http://whqlibdoc.who.int/publications/2011/9789241501750_eng.pdf. Accessed 5th June, 2015. 26158187

[5] Delany-Moretlwe S, Cowan FM, Busza J, Bolton-Moore C, and Fairlie L. Providing comprehensive health services for young key populations: needs, barriers and gaps. Journal of the International AIDS Society 2015, 18(Suppl 1):19833 25724511 10.7448/IAS.18.2.19833 PMC4344539

[6] Hakim AJ, Aho J, Semde G, Diarrassouba M, Ehoussou K, Vuylsteke B, et al The Epidemiology of HIV and Prevention Needs of Men Who Have Sex with Men in Abidjan, Cote d'Ivoire. PLoS One. 2015 4 24;10(4):e0125218. 25909484 10.1371/journal.pone.0125218 PMC4409353

[7] Holland CE, Papworth E, Billong SC, Kassegne S, Petitbon F, Mondoleba V, et al Access to HIV Services at Non-Governmental and Community-Based Organizations among Men Who Have Sex with Men (MSM) in Cameroon: An Integrated Biological and Behavioral Surveillance Analysis. PLoS One. 2015 4 23;10(4):e0122881 10.1371/journal.pone.0122881. eCollection 2015. 25906046 PMC4408025

[8] Hladik W, Barker J, Ssenkusu JM, Opio A, Tappero JW, Hakim A, et al (2012) HIV Infection among Men Who Have Sex with Men in Kampala, Uganda—A Respondent Driven Sampling Survey. PLoS ONE 7(5): e38143 10.1371/journal.pone.0038143 22693590 PMC3364961

[9] Baral S, Sifakis F, Cleghorn F, and Beyrer C. Elevated Risk for HIV Infection among Men Who Have Sex with Men in Low- and Middle-Income Countries 2000–2006: A Systematic Review. PLoS Med, 2007 4(12): p. e339 18052602 10.1371/journal.pmed.0040339 PMC2100144

[10] World Health Organization 2014. Consolidated Guidelines for KAPs Consolidated guidelines on HIV prevention, diagnosis, treatment and care for key populations. 25996019

[11] Ministry of Health Uganda and OR Macro 2012. Uganda AIDS Indicator Survey 2011. health.go.ug/docs/UAIS_2011_REPORT.pdf. Accessed 29, May 2015.

[12] King R, Barker J, Nakayiwa S, Katuntu D, Lubwama G, Bagenda D, et al Men at risk; a qualitative study on HIV risk, gender identity and violence among men who have sex with men who report high risk behavior in Kampala, Uganda. PLoS One. 2013 12 17;8(12):e82937 10.1371/journal.pone.0082937 24358239 PMC3866199

[13] Uganda AIDS Commission 2014. A case for more strategic and increased investment HIV/AIDS Programmes for Uganda 2015–2025

[14] Musinguzi G, Bastiaens H, Matovu JKB, Nuwaha F, Mujisha G, Kiguli J, et al Barriers to condom use among high risk men who have sex with men in Uganda: A qualitative study. PLoS One. 2015 7 14;10(7):e0132297 10.1371/journal.pone.0132297 26172374 PMC4501754

[15] Graneheim U.H. and Lundman B., Qualitative Content analysis in Nursing research: concepts, procedures and measures to achieve trustworthiness, Nurse Education Today, 24, 105–112 14769454 10.1016/j.nedt.2003.10.001

[16] Lewis-Beck M. S., Bryman A., and Liao T. F. (Eds.). (2004). Encyclopedia of social science research methods. (Vols. 1–3). Thousand Oaks, CA: SAGE Publications, Inc. 10.4135/9781412950589

[17] Miles M.B and Huberman A.M., Qualitative data analysis, 2nd Edition, Thousand Oaks, CA:Sage Publications, 1994

[18] Batist E, Brown B, Scheibe A, Baral SD, and Bekker LG. Outcomes of a community-based HIV-prevention pilot programme for township men who have sex with men in Cape Town, South Africa. J Int AIDS Soc, 2013 3(18754): p. 18754. 10.7448/IAS.16.4.18754 PMC3852355 24321116

[19] Geibel Scott, Tun Waimar, Tapsoba Placide, and Kellerman Scott. 2010 “Looking back, moving forward: Understanding the HIV risk and sexual health needs of men who have sex with men, Horizons studies 2001 to 2008,” Horizons Synthesis Background Papers. Washington, DC: Population Council.

[20] Delany-Moretlwe S, Cowan FM, Busza J, Bolton-Moore C, Kelley K, and Fairlie L. Providing comprehensive health services for young key populations: needs, barriers and gaps. J Int AIDS Soc. 2015 2 26;18(2 Suppl 1):19833 10.7448/IAS.18.2.19833. eCollection 2015. 25724511 PMC4344539

[21] Sanders EJ, Graham SM, Okuku HS, van der Elst EM, Muhaari A, Davies A, et al HIV-1 infection in high risk men who have sex with men in Mombasa, Kenya. AIDS. 2007 11 30;21(18):2513–20. 18025888 10.1097/QAD.0b013e3282f2704a

[22] Taegtmeyer M, Davies A, Mwangome M, van der Elst EM, Graham SM, Price MA, et al (2013) Challenges in Providing Counselling to MSM in Highly Stigmatized Contexts: Results of a Qualitative Study from Kenya. PLoS ONE 8(6): e64527 10.1371/journal.pone.0064527 23762241 PMC3676420

[23] van der Elst EM, Gichuru E, Omar A, Kanungi J, Duby Z, Midoun M, et al Experiences of Kenyan healthcare workers providing services to men who have sex with men: qualitative findings from a sensitivity training programme. J Int AIDS Soc. 2013 12 2;16 Suppl 3:18741 10.7448/IAS.16.4.18741 24321109 PMC3852126

[24] Dijkstra M, van der Elst EM, Micheni M, Gichuru E, Musyoki H, Duby Z, et al Emerging themes for sensitivity training modules of African healthcare workers attending to men who have sex with men: a systematic review. Int Health. 2015 5;7(3):151–162. Epub 2015 Jan 16. 10.1093/inthealth/ihu101 25596188 PMC4427535

[25] World Health Organization 2011. Guidelines: prevention and treatment of HIV and other sexually transmitted infections among men who have sex with men and transgender people: recommendations for a public health approach 2011. 26158187

[26] Smit PJ, Brady M, Carter M, Fernandes R, Lamore L, Meulbroek M, et al, HIV-related stigma within communities of gay men: a literature review. AIDS Care., 2012 24(4): p. 405–12. 10.1080/09540121.2011.613910. Epub 2011 Nov 25. 22117138 PMC3379736

[27] Rankin WW, Brennan S, Schell E, Laviwa J, and Rankin SH. The Stigma of Being HIV-Positive in Africa. PLoS Med, 2005 2(8): p. e247 16008508 10.1371/journal.pmed.0020247 PMC1176240

[28] Ross MW, Nyoni J, Larsson M, Mbwambo J, Agardh A, Kashiha J, et al Health care in a homophobic climate: the SPEND model for providing sexual health services to men who have sex with men where their health and human rights are compromised. Glob Health Action 2015, 8: 26096— 10.3402/gha.v8.26096 25787179 PMC4365140

